# Immune-Related Cutaneous Adverse Events Display Distinct Clinical and Molecular Characteristics, Depending on Immune Checkpoints Targeted

**DOI:** 10.3390/cancers17121992

**Published:** 2025-06-14

**Authors:** Lukas Kraehenbuehl, Nicola Winkelbeiner, Patrick Turko, Ramon Staeger, Adhideb Ghosh, Vivienn Kaiser, Pia-Charlotte Stadler, Thierry M. Nordmann, Marie-Charlotte Brüggen, Mitchell P. Levesque, Emmanuel Contassot, Lars E. French, Reinhard Dummer, Barbara Meier-Schiesser

**Affiliations:** 1 Department of Dermatology and Allergology, Kantonsspital Aarau, 5001 Aarau, Switzerland; lukas.kraehenbuehl@ksa.ch (L.K.); reinhard.dummer@ksa.ch (R.D.); 2 Department of Dermatology, University Hospital Zurich, 8091 Zurich, Switzerland; patrick.turko@usz.ch (P.T.); ramon.staeger@usz.ch (R.S.); vivienn.kaiser@usz.ch (V.K.); thierry.nordmann@usz.ch (T.M.N.); marie-charlotte.brueggen@usz.ch (M.-C.B.); mitchell.levesque@usz.ch (M.P.L.); 3 Faculty of Medicine, Dermatology and Venereology, University of Zurich, 8006 Zurich, Switzerland; nicolawinkelbeiner@bluewin.ch (N.W.); adhideb.ghosh@hest.ethz.ch (A.G.); 4 Swim Across America and Ludwig Collaborative Laboratory, Department of Pharmacology, Weill Cornell Medicine, Meyer Cancer Center, New York, NY 10065, USA; 5 Functional Genomics Center Zurich, ETH Zurich and University of Zurich, 8091 Zurich, Switzerland; 6 Department of Proteomics and Signal Transduction, Max Planck Institute of Biochemistry, Martinsried, 82152 Munich, Germany; pr@biochem.mpg.de; 7 Department of Dermatology and Allergy, University Hospital, LMU Munich, 81377 Munich, Germany; lars.french@med.uni-muenchen.de; 8 Department of Biomedicine, University of Basel, 4001 Basel, Switzerland; emmanuel.contassot@unibas.ch; 9 Philip Frost Department of Dermatology, University of Miami, Miami, FL 33136, USA

**Keywords:** immune-related adverse event, immune checkpoint inhibition, cancer immunotherapy, oncodermatology, melanom

## Abstract

**Background/Objectives**: Immune-related cutaneous adverse events (ircAEs) are common complications of cancer immunotherapy and provide insight into immune-related adverse events (irAEs) more broadly. To enhance our molecular understanding, we characterized ircAEs resulting from single-agent (PD1) and combined immunotherapy regimens (P+C). Clinically, maculopapular rash (MPR) and toxic epidermal necrolysis (TEN) resemble ircAEs, providing a valuable basis for investigations. **Methods**: To investigate the transcriptome and immune infiltrates in ircAEs, we conducted transcriptomic analyses and multiplexed immunohistochemistry on skin biopsies from patients receiving PD1 and P+C, as well as those with MPR, TEN, and healthy controls. **Results**: Principal component analysis revealed distinct transcriptomic clustering between ircAEs, MPR, and TEN. Specifically, PD1 ircAEs exhibited a gene expression profile similar to TEN, with upregulation of Type-I-response-related genes (e.g., CXCL9 Log2FC 5.34, *p* < 0.0001, CXCL10 Log2FC 6.03, *p* < 0.0001), while P+C ircAEs more closely resembled MPR. Immune infiltrates differed significantly between all groups (*p* = 0.002 by PERMANOVA for all groups). CD4 T-cells were abundant in the dermis of ircAEs from any type of immunotherapy. However, PD1 stained positive in 1.07% of CD4 cells with PD1 monotherapy, compared to 0.3%, 0.4%, and 0.08% in P+C, MPR, and TEN, respectively. **Conclusions**: This study identified distinct molecular and cellular signatures in ircAEs depending on the type of immune checkpoint blockade. aPD1-associated ircAEs share similarities with the cytotoxic profile of TEN, while P+C more closely mirrored MPR. These findings support the need for tailored management strategies for ircAEs, emphasizing personalized therapeutic approaches to minimize treatment interruptions while preserving the efficacy of cancer immunotherapy.

## Simple Summary

Being the most common immune-related adverse event, immune-related cutaneous adverse events (ircAEs) not only impair patients’ quality of life but also offer critical insights into the broader mechanisms of irAEs. With rapidly increasing numbers of patients receiving immunotherapy, managing these adverse events has never been more important. Our molecular characterization of ircAEs suggests distinct mechanisms depending on the type of immunotherapy, thereby providing a rationale for targeted management. To avoid the pitfalls of broad immunosuppression impairing anti-cancer efficacy, tailored interventions—such as selective biologics—are key. These strategies will be crucial to mitigate ircAEs while maintaining the powerful anti-cancer benefits of immunotherapies.

## 1. Introduction

Targeted blockade of immune checkpoints (ICB) has revolutionized immunotherapeutic approaches. Within the last decades, it enabled impressive clinical results in a rapidly growing number of patients when compared to initial trials by Coley [1]. ICB is now part of recognized standards of care regimens for a multitude of cancer types, such as melanoma, non-melanoma skin cancer, non-small cell lung cancer, renal cell carcinoma, and many others. It has proven to be effective in neoadjuvant, adjuvant, and inoperable settings. Approved indications and those under investigation are still growing, including additional types of cancers, as well as earlier stages, becoming amenable for adjuvant ICB regimens. Ipilimumab (Ipi), a fully human monoclonal IgG1 antibody against cytotoxic T-lymphocyte antigen 4 (CTLA4), was the first approved ICB, demonstrating impressive, durable responses compared to—the standard of care—dacarbazine chemotherapy [2]. Subsequently, targeting of programmed cell death 1 (PD1) and its ligand 1 (PD-L1) was found to provide superior response rates and improved toxicity profiles when compared with Ipi [3,4]. The combination of both CTLA4- and PD1-blockade is more effective yet considerably more toxic than monotherapies [5,6,7]. With multiple additional immune-checkpoints, as well as other immunotherapeutic targets, being under investigation, relatlimab targeting lymphocyte activation gene 3 (LAG-3) has recently been approved for use in combination with PD1-blockade [8].

Up to 90% of ICB-based immunotherapies are complicated by immune-related adverse events (irAEs) [9]. Within those, the skin is the most frequently affected organ [10,11]. Whilst immune-related cutaneous adverse events (ircAEs) rarely are life-threatening, the negative impact on quality of life is considerable, and ICB treatment delays and discontinuation occur regularly [12,13]. Furthermore, due to the ease of accessibility to clinical monitoring and tissue biopsies compared to other organs, ircAEs are frequently considered a “window to irAEs overall”. It has been previously described that ircAEs occur earlier after initiation of ICB regimens than most other irAEs [14,15], although different phenotypes of ircAEs have been found to be associated with different timelines [16].

IrcAEs clinically resemble common adverse cutaneous drug reactions induced by other drugs, such as maculopapular rash, presenting with erythematous macules and papules and frequently associated with a perivascular lymphocytic infiltrate on histology (MPR) and toxic epidermal necrosis (TEN)/ Stevens–Johnson Syndrome (SJS) [17], as well as lichenoid drug reactions [18]. MPR is a type IV hypersensitivity reaction, and with a prevalence of >95%, it is the most frequently occurring adverse cutaneous reaction. Generally, MPR presents in a mild form without organ involvement and is histologically characterized as perivascular inflammation [10,19]. In contrast, TEN/SJS are atypical type 4 hypersensitivity reactions histologically presenting as multiform cytotoxic reactions with organ involvement and rarely occur, with an incidence of 1–2 cases per million per year. Affected patients suffer from severe symptoms, resulting in a mortality rate of 30%. Cutaneous lesions show extensive macules, papules, and/or targetoid lesions. Epidermal necrolysis manifests with varying intensity and presents as detachment of the skin and/or bullous skin lesions frequently, with mucosal involvement [20,21,22,23]. Epidermal necrolysis results from a consequence of pronounced keratinocyte apoptosis driven by cytolytic molecules, such as perforin/granzyme B [24], FasL (CD95L) [24,25] and granulysin [26].

Despite its frequency, the underlying pathomechanisms of ircAEs in response to anti-PD1 treatment remain unclear. It was shown previously, based on gene expression profiling, that certain ircAEs induced by anti-PD1 antibodies resemble TEN rather than MPR or graft-versus-host-disease [27]. In this study, we aim to further characterize skin rashes induced by different immunotherapy targets, e.g., anti-PD1 monotherapy (aPD1) or combined anti-PD1/anti-CTLA4 (P+C) therapy. This is undertaken by systematically comparing both irAEs caused by different immunotherapies and irAEs with the common adverse cutaneous reactions MPR and TEN/SJS using next-generation RNA sequencing and multiplexed immunohistochemistry.

## 2. Materials and Methods

### 2.1. Study Population

Samples for transcriptomic analyses were collected from patients treated with single-agent aPD1 (n = 6) and combined aPD1/aCTLA4 ICB (n = 3) for metastatic melanoma or other advanced skin cancers, as well as from patients with MPR (n = 7), TEN (n = 3), and healthy skin (n = 13) within the department of dermatology at the University Hospital of Zurich, Switzerland. Samples for spatial investigations were identified retrospectively from FFPE skin biopsies available within the department (patient characteristics, Supplementary Table S1). For multiplexed immunohistochemistry, 15 FFPE samples from ircAEs (including 12 aPD1 monotherapy and 3 combined ICB) were identified. As comparators, 4 FFPE samples from healthy skin, 8 from MPR, and 10 from TEN were included. From both aPD1 and MPR groups, 2 samples each failed quality control, with less than 500 cells identified in either the epidermis or dermis and were, therefore, not amenable for analysis.

All patients have signed general research consent and biobanking consent, and this project was approved by the Cantonal Ethics Committee of the Canton of Zurich, Switzerland (KEK) BASEC 2021-00951; approved 5 May 2021 and 2019-01825; approved 11 October 2019.

### 2.2. Sample Processing

For transcriptome analysis, lesional skin biopsy specimens of ircAEs occurring in patients treated with PD1 or P+C from MPR, TEN, and healthy skin were collected under sterile conditions and preserved in RNA later at −20 °C until further use.

### 2.3. RNA Isolation and Sequencing

Samples were thawed, and RNA extraction was carried out using Trizol along with the Qiagen RNAeasy kit (Qiagen, Germany), adhering to the manufacturer’s instructions. For RNA sequencing, total RNA (100–1000 ng per sample) underwent ribosomal RNA depletion, was reverse-transcribed into double-stranded cDNA, and then selectively enriched by polymerase chain reaction (PCR). The enriched libraries’ quality and quantity were assessed using the Fragment Analyzer (Agilent, Santa Clara, CA, USA). Libraries were then normalized to a concentration of 10 nM in Tris-Cl 10 mM, pH 8.5 with 0.1% Tween 20. Sequencing and cluster generation were performed using the HiSeq2500 (Illumina, Inc., San Diego, CA, USA) following the standard protocol. The sequencing dataset is publicly available at the National Institute of Health’s gene expression omnibus (GEO) platform with the accession number GSE297863.

### 2.4. Multiplex Immunohistochemistry

For multiparameter immunohistochemical staining of different immune cell populations, two multiplex panels were developed: a T cell panel, including a cytotoxic marker, and a macrophage panel, including a neutrophil marker; both panels include a keratinocyte marker for the identification of the epidermis.

Staining was performed fully automatically using a Bond RXm autostainer (Leica Biosystems, New York, NY, USA) and the OPAL 7-Color Automation IHC Kit (Akoya Biosciences, Marlborough, MA, USA). The procedure involved deparaffinization, several washes, antigen retrieval with a pH 6 buffer, and blocking with Opal Antibody Diluent/Block. Sequential staining with primary antibodies was conducted for the T cell panel (CD8, FoxP3, IL17A, Granzyme B, PanCK, CD4) and the macrophage panel (pSTAT1, MPO, c-Maf, PanCK, CD68), with dilutions specified in Supplementary Table S2. DAPI was used as a counterstain.

Following primary antibody incubation, the sections were washed again and incubated with Opal Polymer HRP Ms + Rb, followed by the application of Opal fluorescent dyes, diluted as specified. The Tyramide Signal Amplification method enhanced sensitivity, enabling multicolor staining. All materials for multiplexing were ordered from Akoya Biosciences (Akoya Biosciences, Marlborough, MA, USA). For the non-standard Opal 780, additional steps, including TSA-DIG incubation and extra washing, were incorporated. After mounting, the stained slides were scanned using an AKOYA PhenoImager HT (Akoya Biosciences, Marlborough, MA, USA).

### 2.5. Statistical Analysis

Raw sequencing data of the bulk RNA sequencing was processed using the SUSHI framework from the Functional Genomics Center Zurich (FGCZ). Low-quality reads and adapters were trimmed using fastp v0.20 [28]. Filtered reads were aligned to the human reference genome assembly GRCh38.p13 using STAR 2.7.8a. Gene expression levels were quantified using the *featureCounts* program of Rsubread v2.4.3. Batch correction of raw gene counts was performed using the R package RUVseq v1.38. Differential gene expression analysis between different conditions was conducted using the R package edgeR v4.2 [29]. Gene set enrichment analysis was performed utilizing the R package clusterProfiler v4.12 [30] based on gene ontology biological process terms.

Akoya staining images were segmented using adaptive segmentation in InForm version 2.6.0 (Akoya Biosciences, Marlborough, MA, USA). Nuclei were detected using the DAPI channel, and cytoplasmic segments were assumed to form non-overlapping rings around each nucleus. The mean staining intensity for each cellular compartment was output.

These cellular intensity measurements were used to assign each cell a state of “positive” or “negative” separately for each marker by fitting additive mixture models to intensity histograms using the R package mixR [31]. Histograms were transformed using the hyperbolic arcsin transformation when required to obtain accurate model fits, and, depending on the shape of the histogram, we fit either normal, log-normal, or Weibull distributions. We then calculated the probability of belonging to the “positive” distribution for each cell and assigned this state when *p* > 0.95. Finally, each cell was assigned a composite phenotype based on its marker positivity. Cells negative for all markers were assigned the phenotype “other”.

Compositional differences were tested in two ways. First, all cells from all samples of the same subtype were combined into one composition, and differences between these “overall” compositions were analyzed using Fisher’s exact test. Comparisons are made separately for the dermis and the epidermis. Second, to better account for inter-subtype heterogeneity, we performed an ordination analysis common to community ecology. An inter-sample multivariate distance matrix was constructed using the Bray–Curtis dissimilarity. These dissimilarities were visualized using non-metric multidimensional scaling (NMDS). We tested the significance of inter-group dissimilarities using permutational multivariate ANOVA (PERMANOVA). Finally, the contribution of each cell type to the inter-subtype dissimilarities was calculated using the “similarity percentages” method (“simper”). Simper analysis assigns each cell type a magnitude of how strongly it affects the inter-subtype compositional dissimilarity and a *p*-value according to the consistency of the samples. All ordination analyses were performed using the R package “vegan”.

## 3. Results

### 3.1. Expression of Cytotoxicity- and Inflammation-Related Genes in ircAEs Induced by aPD1 Monotherapy Mirrors TEN, Unlike P+C Therapy

**Figure 1:**
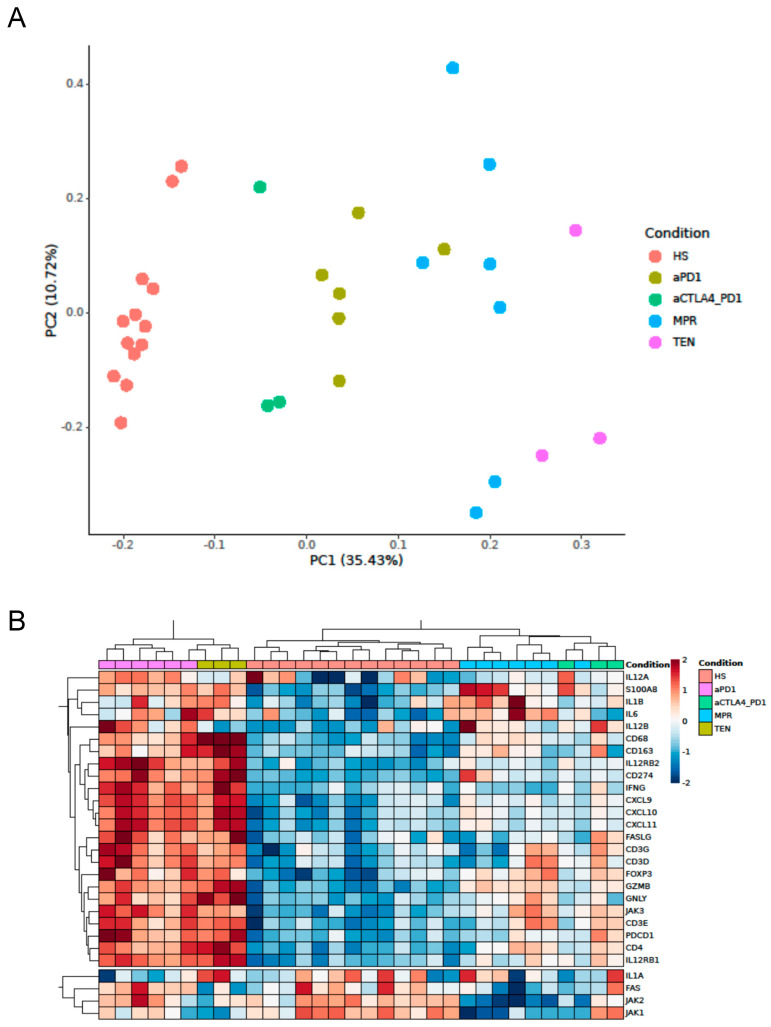

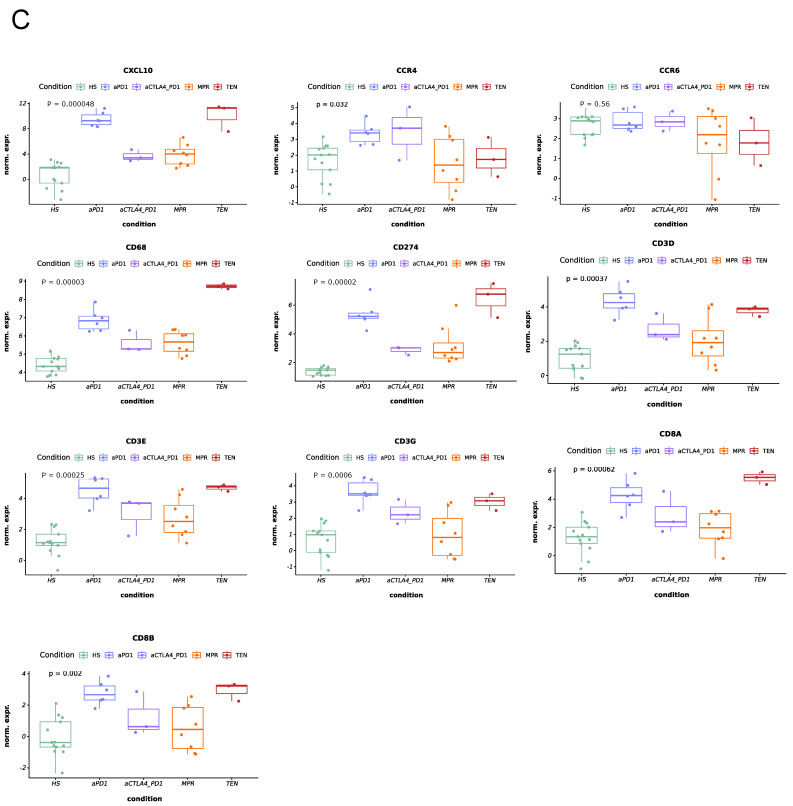
Transcriptomic profiling of ircAEs and common adverse skin reactions. Bulk RNA sequencing was performed on 13 HS, 5 aPD1, 3 aCTLA4_PD1, 3 TEN, and 6 MPR samples. Principal component analysis shows distinct clustering of each disease group (**A**). Illustration of hierarchical clustering (**B**) and box plots (**C**) of selected candidate genes involved in inflammation and cytotoxicity. HS: healthy skin; aPD1: anti-PD1-induced immune-related cutaneous adverse reactions; aCTLA4_PD1: anti-CTLA4/PD1-induced immune-related cutaneous adverse reactions; MPR: maculopapular rash; TEN: toxic epidermal necrolysis.

First, we investigated possible differences related to the immunotherapy target in the transcriptome of ircAEs in response to aPD1 monotherapy versus ircAEs to P+C. Our second comparison was between these ircAEs and non-immunotherapy-related TEN, MPR, and healthy skin. To explore this, we performed bulk RNAseq from the skin biopsies. Principal component analysis (PCA) revealed that the five groups formed distinct clusters, separated by PC1 with 35.43% (Figure 1A), strongly separating healthy from non-healthy skin. Whereas the transcriptomes of both ircAE groups were highly similar to each other, they strongly differed from those of MPR and TEN (Figure 1A). In contrast, analysis of candidate gene transcripts associated with inflammation and cytotoxicity, selected based on our previous findings [27] (including *CXCL10*, *CCR4*, *CCR6*, *CD68*, *CD274*, *CD3D*, *CD3E*, *CD3G*, *CD8A*, *CD8B*), indicated that aPD1 and TEN samples exhibited considerable similarity in immune-related gene expression, while ircAEs from P+C treatment clustered with MPR samples. Healthy skin samples remained largely distinct from all other groups (Figure 1B,C). A list of the top 100 differentially expressed genes can be found in Supplementary Table S3.

### 3.2. IrcAEs Induced by aPD1 Monotherapy Exhibits Stronger Immune Activation Compared to P+C Therapy

**Figure 2:**
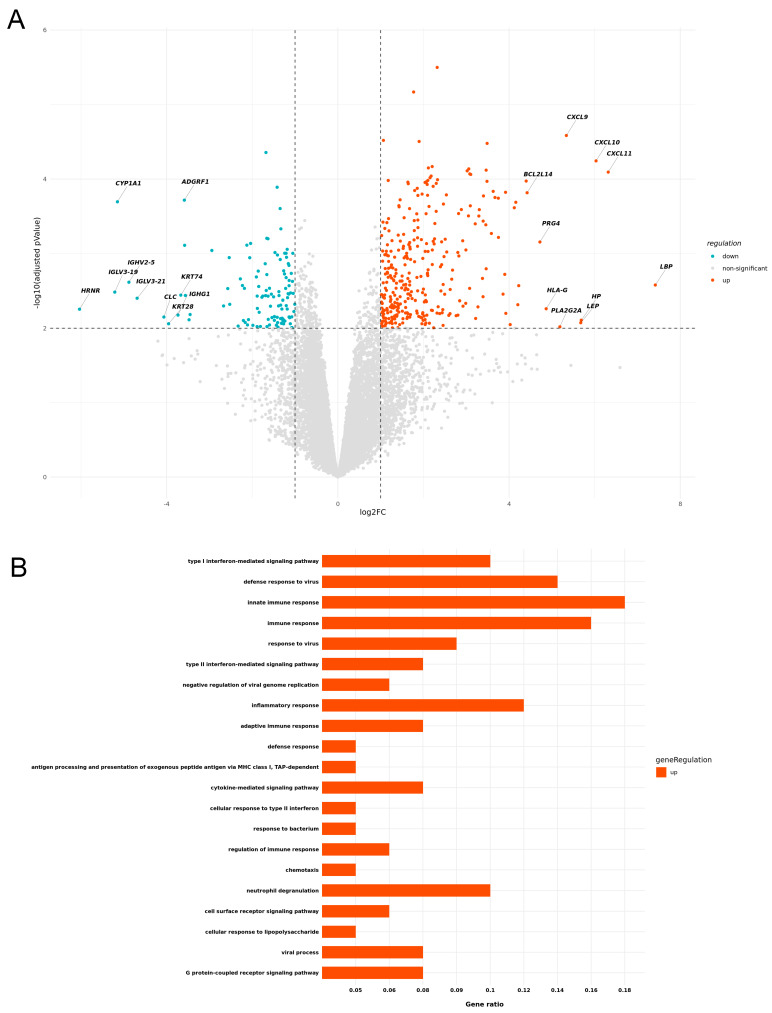
Differential gene expression by immunotherapy regimen and associated pathways. Volcano plot illustrating significantly up- and downregulated genes in aPD1 over aCTLA4_PD1 (**A**). Gene enrichment analysis of biological processes (adjusted *p*-value < 0.01) displays significantly upregulated pathways in aPD1 compared to aCTLA4_PD1 (**B**).

The direct comparison of aPD1 ircAEs and aPD1/CTLA4 ircAEs revealed 324 significantly upregulated and 114 downregulated transcripts (log2FC > 1; *p*-value < 0.01) in aPD1 ircAEs (Figure 2A). The most significantly upregulated transcripts included those coding for Th1 chemokines, such as *CXCL9* (Log2FC 5.34, *p* < 0.0001), *CXCL10* (Log2FC 6.03, *p* < 0.0001), and *CXCL11* (Log2FC 6.32, *p* < 0.0001).

Based on our previous findings of resemblance between TEN and aPD1 [27], we specifically investigated distinctive characteristics between these two groups. Over-representation analysis of significantly upregulated genes (adjusted *p*-value < 0.01) revealed a marked upregulation of most significant biological processes associated broadly with various immune responses, including innate immune response, neutrophil degranulation, and inflammatory response, in TEN compared to aPD1 lesional skin. In contrast, aPD1 ircAE lesions exhibited a strong upregulation of type I interferon-associated genes compared to P+C ircAEs, alongside several other inflammatory processes and immune responses to infection (Figure 2B; Supplementary Figure S1).

### 3.3. Spatial Assessment of Immune Infiltrates Reveals Distinct Cell Population Patterns in ircAEs and Cutaneous Adverse Drug Reactions

**Figure 3:**
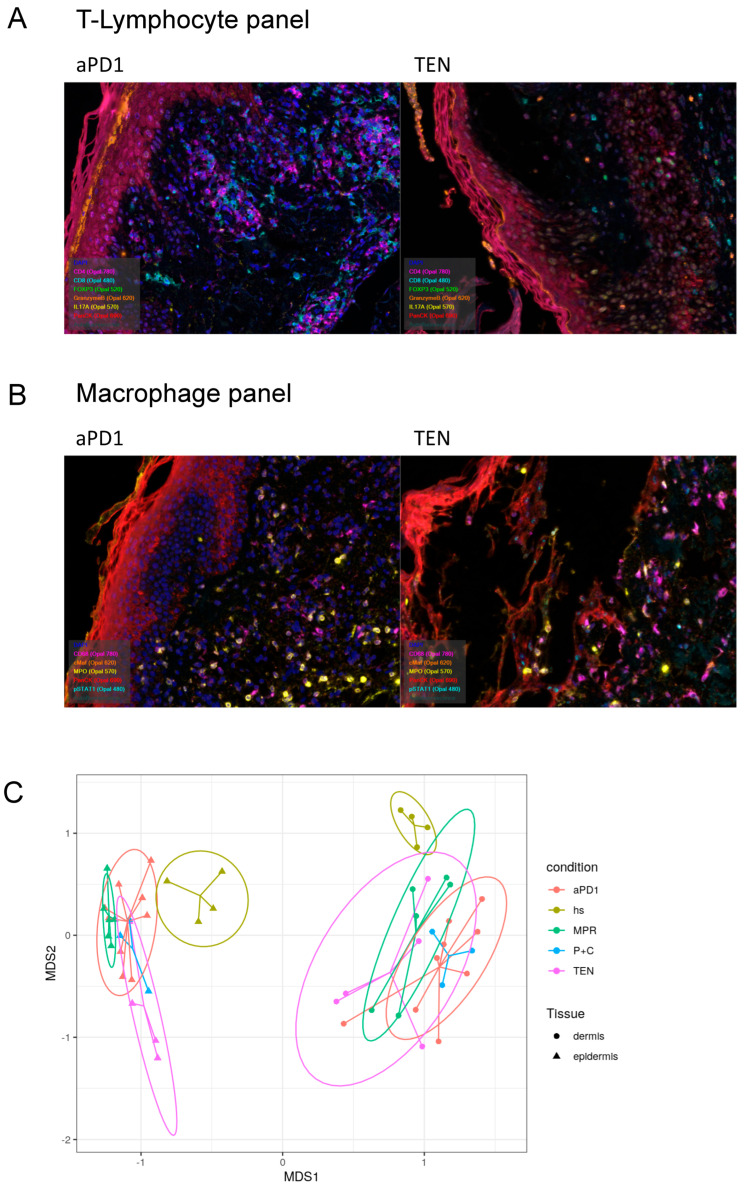

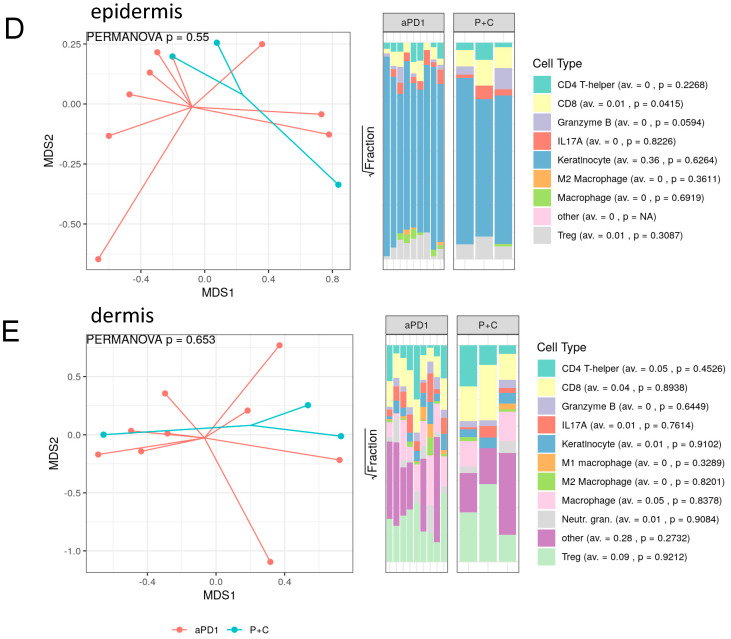
Distribution of immune compartments by type of skin reaction. Multiplex immunohistochemistry staining based on two different immune panels dedicated to lymphocytes and macrophages was performed on FFPE samples of non-lesional skin of 4 HS and lesional skin of 15 patients suffering from ircAEs (including 12 aPD1 and 3 aCTLA4_PD1), 8 MPR, and 10 TEN patients using the Akoya system. Representative staining for T-lymphocyte panel (**A**) and macrophage panel (**B**). Staining was performed with antibodies identifying various cell types, including CD4+ and CD8+ T cells, regulatory T cells (Tregs), Th17 cells, cells expressing the cytotoxicity marker Granzyme B, macrophages (both M1 and M2 types), neutrophilic granulocytes, and keratinocytes. Relative quantification of cell types by condition in the dermis (**left**) and epidermis (**right**). Inter-sample multivariate “distance” scores were calculated using the compound in non-metric multidimensional scaling (NMDS) using the Bray–Curtis dissimilarity index and distances were visualized through non-metric multidimensional scaling (**C**). “Similarity percentages” (simper) analysis was conducted to detect which cell types contribute to how much variation in the epidermis (**D**) and dermis (**E**) of aPD1 vs. P+C samples (**left**). The square root fraction of cell type distribution and associated *p*-values are indicated (**right**). PERMANOVA was used for statistical analysis. HS: healthy skin; aPD1: anti-PD1-induced immune-related cutaneous adverse reactions; P+C: anti-CTLA4/PD1-induced immune-related cutaneous adverse reactions; MPR: maculopapular rash; TEN: toxic epidermal necrolysis.

To differentiate ircAEs induced by aPD1 monotherapy or P+C therapy from TEN and MPR based on cell type composition, multiplex immunohistochemical staining was conducted on FFPE samples of non-lesional skin of four healthy donors and lesional skin of fifteen patients suffering from ircAEs (including twelve aPD1 monotherapy and three combined ICB), eight MPR, and ten TEN patients using the Akoya system. Staining was performed with antibodies identifying key cell types, including CD4^+^ and CD8^+^ T cells, regulatory T cells (Tregs), Th17 cells, cells expressing the cytotoxicity marker Granzyme B, macrophages (both M1 and M2 types), neutrophilic granulocytes, and keratinocytes (Figure 3A, Supplementary Figure S4).

We first investigated distinct cell type characteristics among the different disease groups and healthy controls using inter-sample multivariate “distance” scores using the Bray–Curtis dissimilarity index (Figure 3C–E, Supplementary Figure S2). A PERMANOVA to test for overall differences among the groups yielded highly significant results (*p* = 0.002).

Subsequently, we explored pairwise differences for each analyzed patient sample group. These comparisons (Supplementary Figure S2) revealed that all disease groups significantly differ in their cellular composition from healthy skin. Among the disease groups, significant differences in cellular compositions were observed only between aPD1 ircAEs and MPR (*p* = 0.03).

The dissimilarity between TEN and healthy skin (*p* = 0.009) was predominantly driven by the numbers of CD8+ T cells (*p* = 0.0025), macrophages (*p* = 0.0049), and Tregs (*p* = 0.0019). The cell types contributing most to the dissimilarity between MPR and healthy skin (*p* = 0.007) were macrophages (*p* = 0.0046) and neutrophils (*p* = 1 × 10^−4^). ircAEs induced by aPD1 ICB also significantly differed from healthy overall (*p* = 0.004); however, no statistically significant differences in single cell types were detected. Conversely, the dissimilarity in cell type composition in irAEs caused by combination ICB was mainly due to strong differences in CD8+ T cell numbers (*p* = 4 × 10^−4^) and Tregs (*p* = 0.0391) (Figure 3D,E).

When comparing the different disease groups, no overall significant differences in dermal cell composition were detected. However, analysis of each cell type revealed significantly higher amounts of IL-17A-positive T cell (CD4/CD8) dermal cells in TEN compared to MPR (*p* = 0.0373), while neutrophils were higher in MPR (*p* = 0.0467) (Supplementary Figure S2). The cell composition of both ircAE groups did not significantly differ from each other in the dermis (Figure 3E); however, a significantly higher abundance of CD8+ T cells was noted in the epidermis of P+C samples compared to aPD1 ircAEs (Figure 3D).

**Figure 4:**
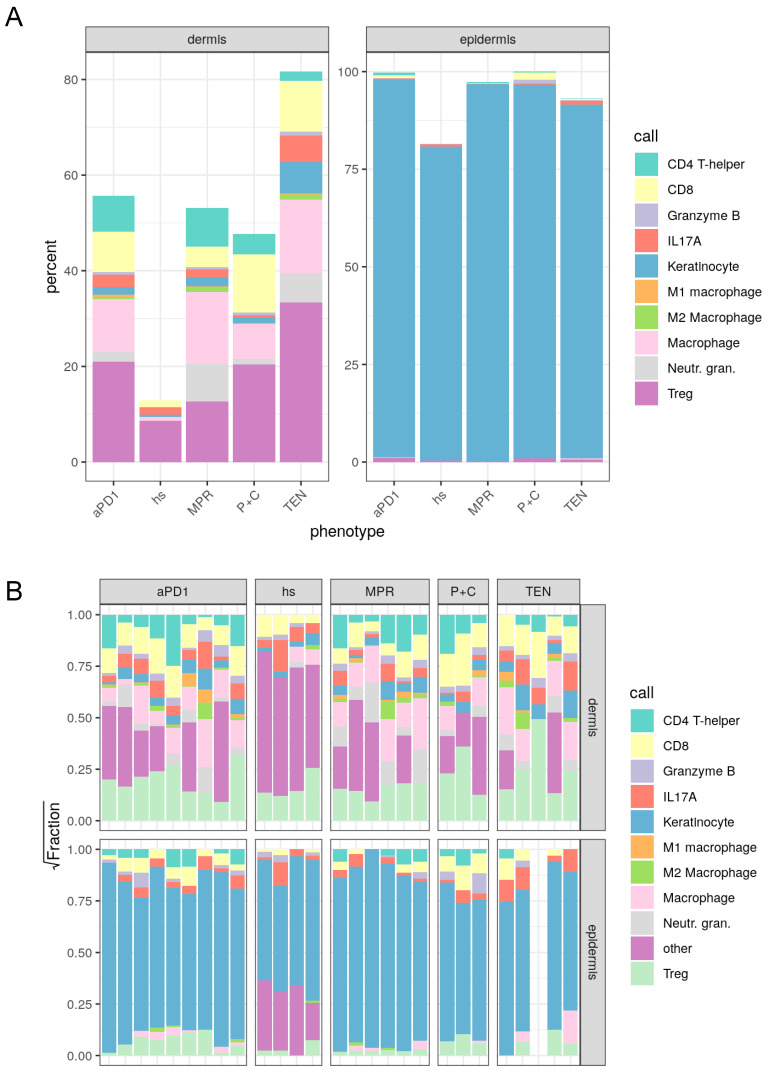
Spatial analysis of immune compartment. Multiplex immunohistochemistry staining based on two different immune panels dedicated to lymphocytes and macrophages was performed on FFPE samples of non-lesional skin of 4 HS and lesional skin of 15 patients suffering from ircAEs (including 12 aPD1 and 3 aCTLA4_PD1), 8 MPR, and 10 TEN patients using the Akoya system (see also Figure 3). (**A**) Relative quantification of cell types by condition in the dermis (**left**) and epidermis (**right**). (**B**) Relative square root fractions of cell types in the dermis (**upper**) and epidermis (**lower**). HS: healthy skin; aPD1: anti-PD1-induced immune-related cutaneous adverse reactions; aCTLA4_PD1: anti-CTLA4/PD1-induced immune-related cutaneous adverse reactions; MPR: maculopapular rash; TEN: toxic epidermal necrolysis.

As a next step, a detailed analysis of the cell type composition was performed. Total dermal macrophages showed the highest abundance in TEN (19%) compared to all other disease groups (MPR: 10.6%, aPD1: 7.6%, P+C: 6.7%) and were even much higher in numbers in the epidermis of TEN (1.59%) compared to the other disease groups (MPR: 0.1%, aPD1 0.27%, P+C 0.04%) (Table 1, Figure 4A). Furthermore, IL-17A^+^ T cells were higher in number in the dermis of TEN (5.4%) compared to the other disease groups, with ircAEs induced by aPD1 showing the second highest abundance (MPR 1.9%, aPD1 2.6%, P+C 0.4%, healthy 1.5%). Total CD4+ cells in the dermis showed the highest abundance in MPR (9.2%) and aPD1-induced ircAEs (7.9%; TEN: 1.5%, P+C: 4.1%, healthy: 0%). Interestingly, epidermal CD4+ cells were strongly elevated in aPD1 ircAEs (1.07%) compared to the other disease groups (TEN 0.08%, MPR 0.4%, P+C 0.3%) and healthy skin (0%), indicating higher exocytosis. In addition, Tregs were highest in number in aPD1 ircAEs and TEN of all disease groups both in the dermis (aPD1 ircAEs: 22.1%, TEN: 13.8%) and epidermis (aPD1 ircAEs: 1.3%, TEN: 1.3%) (Table 1 and Table 2, Figure 4A,B, Supplementary Figure S3).

## 4. Discussion

In the present investigation, we have identified molecular resemblance of ircAEs occurring in combined immunotherapy with aCTLA4 and aPD1 agents to MPR and TEN with ircAEs occurring with aPD1 monotherapy, respectively. Supporting our previous findings [27], samples from ircAEs triggered by aPD1 monotherapy showed clinical correspondence to a lichenoid phenotype and a striking resemblance to specimens from patients with TEN. Overall, the pattern aligns with a cytotoxic reaction. Importantly, ircAEs differ significantly from healthy skin, both in their transcriptome and immune cell infiltration. Upon further analysis, distinct characteristics were identified for ircAEs caused by aPD1 monotherapy and by the P+C. While ircAEs due to aPD1 resemble TEN, P+C showed considerable overlap with samples from MPR.

Our current findings are consistent with our initial observation that lichenoid ircAEs, due to aPD1, resemble TEN and exhibit a cytotoxic phenotype; however, we were able to expand beyond this observation to describe a similar resemblance between MPR and ircAEs in combined immunotherapy. Importantly, we describe immunologic and histomorphologic resemblance between ircAEs and other skin rashes, including maculopapular drug eruption and toxic epidermal necrolysis. Interestingly, despite ircAEs due to aPD1 resembling TEN—a life-threatening dermatologic condition—more closely, the clinical appearance of ircAEs with aPD1 is comparable to or less severe than that occurring with P+C.

Whereas ircAEs of the maculopapular phenotype are more strongly associated with aCTLA4 monotherapy or P+C, lichenoid ircAEs have been prototypically associated with aPD1 immunotherapy. However, multiple other types of ircAEs, including pruritus on seemingly unchanged skin and eczematous and psoriasiform ircAEs, have not been attributed to a specific ICB target to date. The molecular characteristics identified in this investigation may, therefore, depend both on the immunologic effect of the targeted immune checkpoint and the clinical appearance of the rash. Meanwhile, other types of ircAEs might be attributable to shared mechanisms induced by blocking any immune checkpoint. Mechanistic investigations into immune-related adverse events and, in particular, into ircAEs have proven difficult due to the lack of optimal murine models [27]. Therefore, a comparative deep characterization of ircAEs secondary to the blockade of different immune checkpoints provides valuable insight into underlying mechanisms. Importantly, comparative studies of ircAEs across different ICI targets, as well as similarities with other skin conditions, are crucial for rationally selecting investigational targeted treatments for future trials and challenging patient cases. Furthermore, focusing on aPD1 against P+C comparisons is highly relevant, as these two regimens are the most widely used in immunotherapy and are likely to remain the cornerstone of ICI treatments in the foreseeable future. Management of ircAEs remains challenging [32]. In addition to rapidly resolving symptoms, maintaining optimal oncological outcomes depends on minimizing treatment interruptions. However, continuing ICB therapy can worsen or maintain ircAEs. While systemic corticosteroids are typically effective, higher doses and prolonged use are likely to impair ICB’s efficacy [32]. A more detailed molecular and cellular characterization of ircAEs is, therefore, warranted to enable targeted interventions.

To prevent interruptions and discontinuation of ICB-based cancer treatments due to irAEs, several mitigation strategies have been proposed. These include the management of irAEs and preemptive immune modulation for patients with preexisting auto-immune conditions, such as with interleukin 6 blockade [32]. Multiple immunomodulators have been investigated for the management of ircAEs, including dupilumab [33] and omalizumab [34], amongst others. Of note, no controlled trials have yet assessed targeted biologic interventions for mitigating ircAEs in terms of overall safety, efficacy on ircAEs, and their impact on cancer outcomes.

Conducting such trials is crucial, though challenging, due to patient settings and the large sample sizes required. Thus, the most promising approaches should be prioritized, which, in our opinion, would involve optimal patient selection based on clinical and potential molecular characteristics of individual ircAEs.

In a recent large prospective investigation, we have identified striking molecular differences between different ircAE phenotypes [35]. IFN-gamma mRNA levels were highest in the lichenoid phenotype, while IL-17A was elevated in the maculopapular phenotype. Notably, MPR-type ircAEs were strongly associated with P+C, whereas lichenoid ircAEs were predominantly linked to aPD1 monotherapy [35]. These findings, along with others, provide a foundation for optimal treatment selection.

In line with our recent investigation [36] comparing immune-related lichen planus (irLP) with sporadic LP, lymphocyte exocytosis into the epidermis was significantly more abundant in patients with ircAEs to aPD1 compared to P+C ircAEs, as well as TEN and MPR comparators. Interestingly, patients with ircAEs, following aPD1/CTLA4 combination, have higher relative abundances of CD8 lymphocytes in both the dermis and epidermis when compared to those following aPD1 monotherapy. Intriguingly and in line with our recent findings [36], ircAEs to aPD1 monotherapy were characterized by increased expression of genes related to interferon-gamma, also termed cytotoxic genes (Figure 2A). In conclusion, we demonstrate molecular similarities between ircAEs occurring in the context of P+C and MPR, in addition to previously observed overlaps between ircAEs due to aPD1 monotherapy and TEN. Our investigation supports a differentiated approach to ircAE management based on clinical factors such as the type of ICB and the clinical presentation, complemented by thorough histopathological and molecular analyses. This high-resolution assessment of individual ircAEs can facilitate tailored treatment approaches, improving patient quality of life while preserving maximal anti-tumor efficacy.

## 5. Conclusions

Based on our findings, targeting IFN signaling in lesions caused by aPD1 monotherapy appears to be a promising therapeutic direction. However, as systemic inhibition of IFN pathways may promote tumor growth, the use of topical JAK inhibitors is a highly promising alternative. This localized intervention (ongoing clinical trial BASEC 2023-01779) could provide the dual benefit of mitigating ircAEs while preserving the desired anti-tumor effects of ICB therapy.

## Figures and Tables

**Table 1:** Raw percentages of cell types in the dermis.

	hs	aPD1	P+C	MPR	TEN
CD4 T-helper	0.0000000	7.8935707	4.1027254	9.1723890	1.4977364
CD8	1.4211965	8.7100173	11.6251376	4.6560604	12.3676716
Granzyme B	0.0971758	0.6571705	0.5572157	0.5788885	0.4311665
IL17A	1.5851807	2.6123781	0.3999371	1.9213111	5.3736966
Keratinocyte	0.3826298	1.6830588	1.0515200	2.2456883	5.1785370
Ml macrophage	0.0000000	0.8402752	0.7010133	0.1317470	0.4311665
M2 macrophage	0.1457637	0.4612456	0.0943672	1.2426142	1.7473591
Macrophage	1.5669602	10.6953243	10.4410542	17.5712632	19.2549896
Neutr. gran.	0.1457637	2.1646494	1.5233559	9.4169195	1.9459226
Treg	8.5089584	22.1494046	19.5564743	14.6718301	37.8110356
Other	86.1463711	42.1329056	49.9471993	38.3912887	13.9607185

**Table 2:** Raw percentages of cell types in the epidermis.

Call	hs	aPD1	P+C	MPR	TEN
CD4 T-helper	0.0000000	1.0734042	0.2959414	0.4121304	0.0795281
CD8	0.0795042	0.7045840	1.7051860	0.1803070	0.7157532
Granzyme B	0.1734637	0.3293258	1.6065389	0.0901535	0.6362251
IL17A	0.6017021	0.3091041	0.2113867	0.1442456	1.9285572
Keratinocyte	80.1618994	95.9794184	95.1803833	98.9585122	93.7239048
M2 macrophage	0.0216830	0.0259994	0.0000000	0.0257581	0.0000000
Macrophage	0.0000000	0.2729938	0.0422773	0.1030326	1.5905627
Treg	0.5637569	1.3051703	0.9582864	0.0858605	1.3254689
Other	18.3979907	0.0000000	0.0000000	0.0000000	0.0000000

## Data Availability

De-identified data can be made available upon reasonable request to the corresponding author.

## Supplementary Materials

The following supporting information can be downloaded at: https://www.mdpi.com/article/10.3390/cancers17121992/s1, Table S1: Patient characteristics. Table S2: Dilution of antibodies and assigned Opal fluorophores. Table S3: Top 100 differentially expressed genes. Figure S1: Differential gene expression by pathway. Figure S2: Pair-wise comparisions of Immune infiltrates. Figure S3: Detailed composition of immune compartments. Figure S4: Spatial analysis of immune compartment.

## Abbreviations

The following abbreviations are used in this manuscript:

ircAEs	immune-related cutaneous adverse events
irAEs	immune-related adverse events
P+C	combined immunotherapy regimen
MPR	maculopapular rash
TEN	toxic epidermal necrolysis
PD1	programmed cell death 1
PD-L1	programmed cell death 1 ligand
CTLA4	cytotoxic T-lymphocyte antigen 4
KEK	Cantonal Ethics Committee of the Canton of Zurich, Switzerland
