# Supplementary material for: Immune-Related Cutaneous Adverse Events Display Distinct Clinical and Molecular Characteristics, Depending on Immune Checkpoints Targeted
Source: Cancers (Basel). 2025 Jun 14;17(12):1992. doi: 10.3390/cancers17121992 (PMC12190265; doi:10.3390/cancers17121992)
Supplement: Supplementary file 1 [file cancers-17-01992-s001.zip › Figures S1-S4.pptx]

## Slide 1
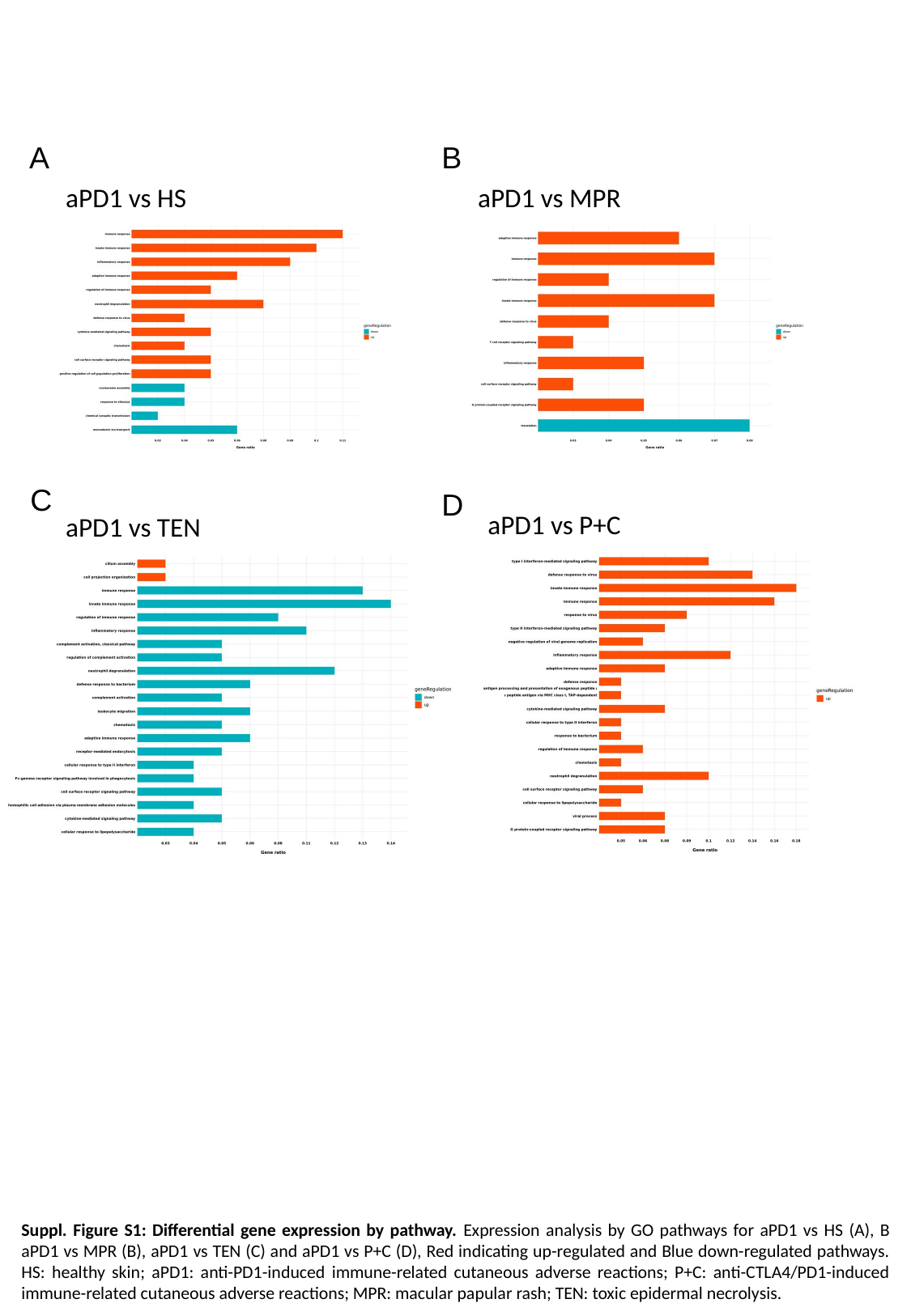

A
B
aPD1 vs HS
aPD1 vs MPR
C
D
aPD1 vs P+C
aPD1 vs TEN
Suppl. Figure S1: Differential gene expression by pathway. Expression analysis by GO pathways for aPD1 vs HS (A), B aPD1 vs MPR (B), aPD1 vs TEN (C) and aPD1 vs P+C (D), Red indicating up-regulated and Blue down-regulated pathways. HS: healthy skin; aPD1: anti-PD1-induced immune-related cutaneous adverse reactions; P+C: anti-CTLA4/PD1-induced immune-related cutaneous adverse reactions; MPR: macular papular rash; TEN: toxic epidermal necrolysis.

## Slide 2
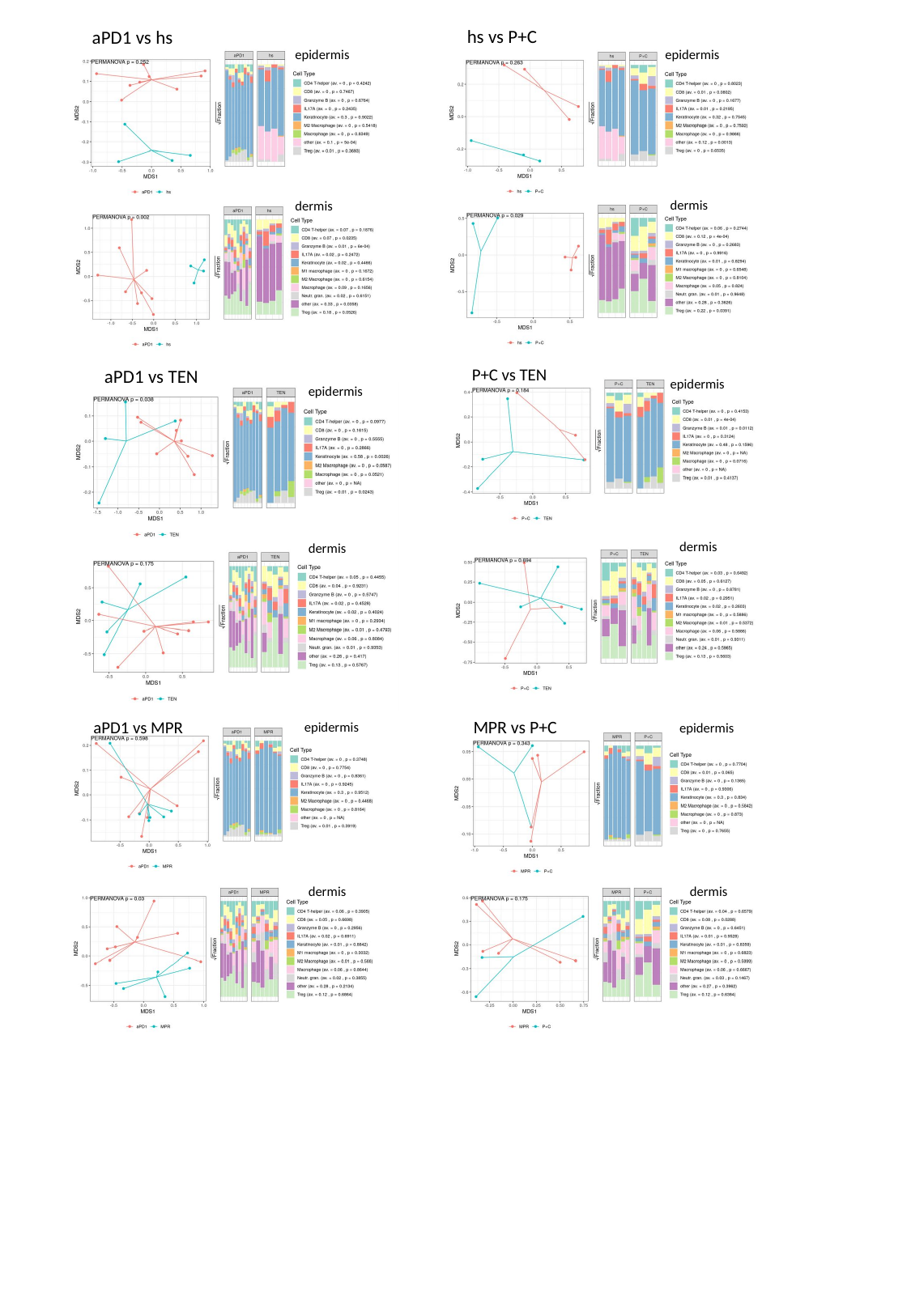

hs vs P+C
aPD1 vs hs
epidermis
epidermis
dermis
dermis
P+C vs TEN
aPD1 vs TEN
epidermis
epidermis
dermis
dermis
MPR vs P+C
aPD1 vs MPR
epidermis
epidermis
dermis
dermis

## Slide 3
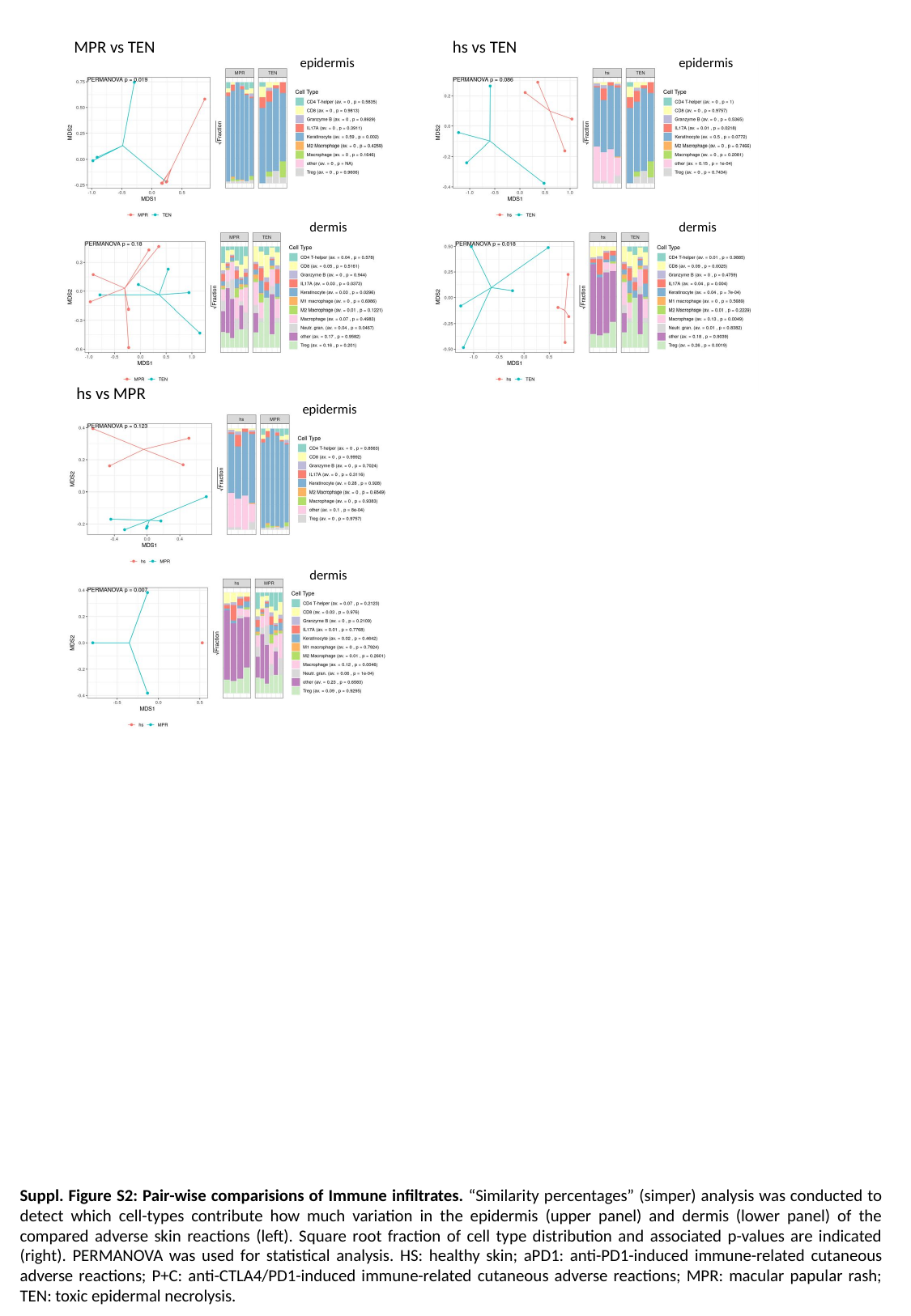

MPR vs TEN
hs vs TEN
epidermis
epidermis
dermis
dermis
hs vs MPR
epidermis
dermis
Suppl. Figure S2: Pair-wise comparisions of Immune infiltrates. “Similarity percentages” (simper) analysis was conducted to detect which cell-types contribute how much variation in the epidermis (upper panel) and dermis (lower panel) of the compared adverse skin reactions (left). Square root fraction of cell type distribution and associated p-values are indicated (right). PERMANOVA was used for statistical analysis. HS: healthy skin; aPD1: anti-PD1-induced immune-related cutaneous adverse reactions; P+C: anti-CTLA4/PD1-induced immune-related cutaneous adverse reactions; MPR: macular papular rash; TEN: toxic epidermal necrolysis.

## Slide 4
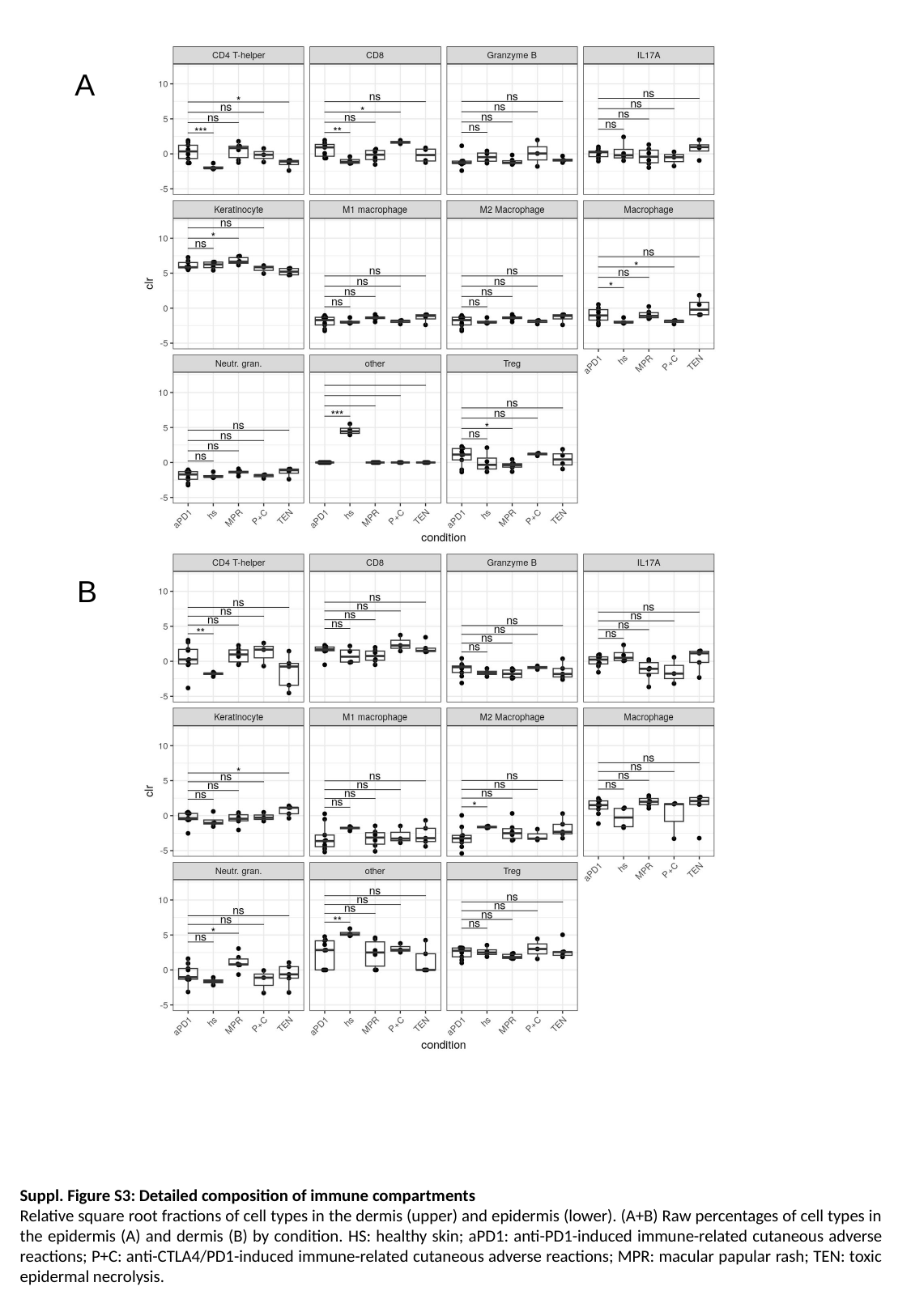

A
B
Suppl. Figure S3: Detailed composition of immune compartments
Relative square root fractions of cell types in the dermis (upper) and epidermis (lower). (A+B) Raw percentages of cell types in the epidermis (A) and dermis (B) by condition. HS: healthy skin; aPD1: anti-PD1-induced immune-related cutaneous adverse reactions; P+C: anti-CTLA4/PD1-induced immune-related cutaneous adverse reactions; MPR: macular papular rash; TEN: toxic epidermal necrolysis.

## Slide 5
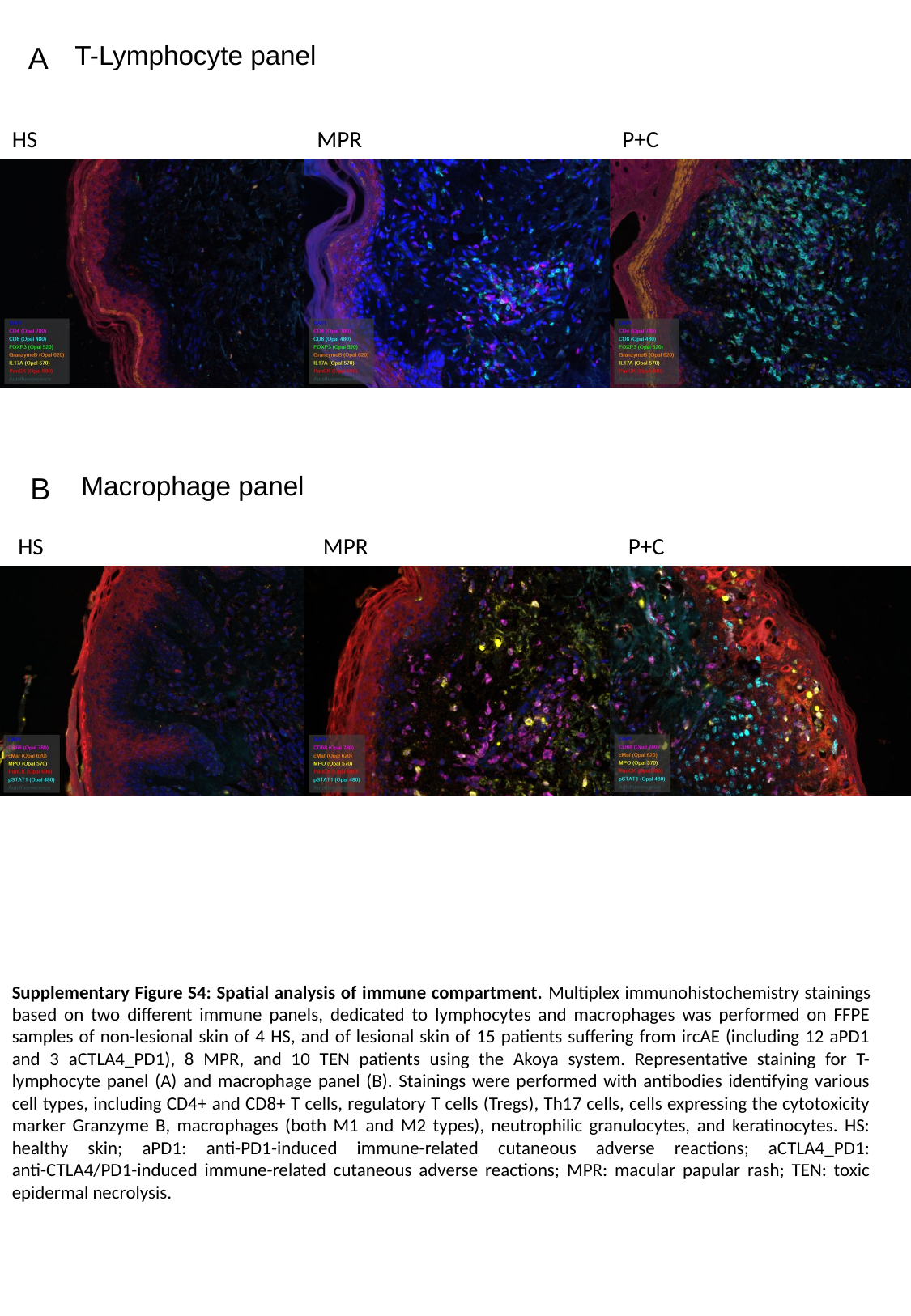

T-Lymphocyte panel
A
MPR
P+C
HS
Macrophage panel
B
MPR
P+C
HS
Supplementary Figure S4: Spatial analysis of immune compartment. Multiplex immunohistochemistry stainings based on two different immune panels, dedicated to lymphocytes and macrophages was performed on FFPE samples of non-lesional skin of 4 HS, and of lesional skin of 15 patients suffering from ircAE (including 12 aPD1 and 3 aCTLA4_PD1), 8 MPR, and 10 TEN patients using the Akoya system. Representative staining for T-lymphocyte panel (A) and macrophage panel (B). Stainings were performed with antibodies identifying various cell types, including CD4+ and CD8+ T cells, regulatory T cells (Tregs), Th17 cells, cells expressing the cytotoxicity marker Granzyme B, macrophages (both M1 and M2 types), neutrophilic granulocytes, and keratinocytes. HS: healthy skin; aPD1: anti-PD1-induced immune-related cutaneous adverse reactions; aCTLA4_PD1: anti-CTLA4/PD1-induced immune-related cutaneous adverse reactions; MPR: macular papular rash; TEN: toxic epidermal necrolysis.
