# Supplementary material for: Immune-Related Cutaneous Adverse Events Display Distinct Clinical and Molecular Characteristics, Depending on Immune Checkpoints Targeted
Source: Cancers (Basel). 2025 Jun 14;17(12):1992. doi: 10.3390/cancers17121992 (PMC12190265; doi:10.3390/cancers17121992)
Supplement: Supplementary file 1 [file cancers-17-01992-s001.zip › Tables S1 and S2.pdf]

Supplementary Table S1. Patient characteristics.

Pat no.	sex	age at event	Triggering immunotherapy	specific Immunotherapy	Tumor type and stage	Start iT until appearance rash	Start IT until biopsy	Histological diagnosis	pretherapy	Technique
**1**	female	19	Mono Anti-PD-1	Cemiplimab	Xeroderma pigmentosum; cutaneous carcinogenesis with multiple BCCs and SCCs	10 d	11 d	lichenoid drug exanthema	none	RNAseq
**2**	male	49	Kombi Anti-PD-1 & Anti-CTLA-4	Ipilimumab/Nivolumab	Metastatic melanoma stage IV (pT2a, cN3c, M1a)	48 d	48 d	Drug reaction with lichenoid aspects	Mono Anti-PD-1 (Pembrolizumab)	RNAseq
**3**	male	56	Kombi Anti-PD-1 & Anti-CTLA-4	Ipilimumab/Nivolumab	Metastatic melanoma stage IV (pT4b N3b M1a)	280 d	280 d	lichenoid reaction with concomitant spongiosis	none	RNAseq
**4**	male	73	Mono Anti-PD-1	Pembrolizumab	uveal melanoma stage IV (pT3aN0M1c)	40 d	42 d	Drug reaction	none	RNAseq
**5**	female	49	Kombi Anti-PD-1 & Anti-CTLA-4	Ipilimumab/Nivolumab	Metastatic melanoma, stage IV (pT3a N0(sn0/3) M1a)	7 d	7 d	lichenoid drug exanthema	none	RNAseq
**6**	male	66	Mono Anti-PD-1	Pembrolizumab	Metastatic melanoma stage IV	43 d	63 d	lichenoid drug reaction	Mono Anti-CTLA-4 (Ipilimumab)	RNAseq
**7**	male	76	Mono Anti-PD-1	Pembrolizumab	Metastatic melanoma stage IV (pT4 pN3 cM1)	3 d	10 d	TEN	Mono Anti-CTLA-4 (Ipilimumab)	RNAseq
**8**	male	81	Mono Anti-PD-1	Pembrolizumab	Metastatic melanoma stage IV (pT4, N2c, M1c)	2 d	2 d	Focal cytotoxic reaction under PD-1 therapy	Mono Anti-CTLA-4 (Ipilimumab)	RNAseq
**9**	male	73	Mono Anti-PD-1	Pembrolizumab	Metastatic melanoma stage IV (pT1 N1b M1c)	359 d	380 d	Pityriasis lichenoides in the context of anti-PD-1	Mono Anti-CTLA-4 (Ipilimumab)	RNAseq
**10**	female	74	Mono Anti-PD-1	Pembrolizumab	Metastatic melanoma stage IV (cT4a, pN1b, cM1b), palliative	327 d	327 d	Lichenoid reaction with compact keratinization, hypergranulosis, acanthosis and lichenoid changes in the area of the junctional zone	none	mIHC
**11**	male	80	Mono Anti-PD-1	Pembrolizumab	Metastatic melanoma stage IV (pTxN0M1b)	343 d	373 d	Drug reaction	Mono Anti-CTLA-4 (Ipilimumab)	mIHC
**12**	male	75	Mono Anti-PD-1	Pembrolizumab	Metastatic melanoma stage IV (pT3bN3M1b)	541 d	542 d	Lichenoid dermatitis	Mono Anti-CTLA-4 (Ipilimumab)	mIHC
**13**	female	56	Mono Anti-PD-1	Pembrolizumab	Metastatic melanoma stage IIIC (pT4b, pN1a(1/2 sn), cM0), adjuvant therapy	230 d	259 d	lichenoid reaction under pembrolizumab	none	mIHC
**14**	female	73	Mono Anti-PD-1	Nivolumab	Metastatic melanoma stage IIIB pT1b N1b M0	294 d	294 d	Lichenoid reaction, compatible with lichen planus-like keratosis	none	mIHC
**15**	male	46	Mono Anti-PD-1	Pembrolizumab	Metastatic melanoma stage IIIC (T2a, N3, M0)	1075 d	1076 d	lichenoid dermatitis	none	mIHC
**16**	male	74	Mono Anti-PD-1	Nivolumab	Metastatic melanoma stage IV (pT3b, pN1a, cM1d)	319 d	378 d	Lichenoid drug reaction	Kombi Anti-PD-1 & Anti-CTLA-4 (Ipilimumab/Nivolumab	mIHC
**17**	male	53	Mono Anti-PD-1	Pembrolizumab	Metastatic melanoma stage IIIC (pT3b, N2c, cM0)	261 d	262 d	Lichenoid inflammation	none	mIHC
**18**	male	69	Kombi Anti-PD-1 & Anti-CTLA-4	Ipilimumab/Nivolumab	Metastatic melanoma stage IV (pT3b, pN2b, cM1c)	109 d	109 d	Interface dermatitis, primarily compatible with a drug reaction	Mono Anti-PD-1 (Nivolumab)	mIHC

**19**	male	60	Mono Anti-PD-1	Nivolumab	Metastatic melanoma stage IV (pT2a N2c M1d)	25 d	28 d	Interface dermatitis, primarily compatible with a lichenoid drug reaction	Kombi Anti-PD-1 & Anti-CTLA-4 (Ipilimumab/Nivolumab	mIHC
**20**	male	71	Mono Anti-PD-1	Pembrolizumab	Metastatic uveal melanoma stage IV	88 d	88 d	Typical lichenoid and spongiotic inflammation pattern	Mono Anti-CTLA-4 (Ipilimumab)	mIHC
**21**	male	65	Kombi Anti-PD-1 & Anti-CTLA-4	Ipilimumab/Nivolumab	Metastatic melanoma stage IIIC pT3b N2c (1/1) M0	45 d	45 d	Lichenoid reaction, well compatible with typical exanthema under anti-PD-1 therapy	none	mIHC
**22**	male	66	Mono Anti-PD-1	Pembrolizumab	Metastatic melanoma stage IV	43 d	63 d	Well compatible with pityriasis lichenoides chronica resp. a lichenoid drug reaction with follicular accentuation under an anti-PD1 antibody	Mono Anti-CTLA-4 (Ipilimumab)	mIHC
**23**	female	80	Mono Anti-PD-1	Pembrolizumab	Metastatic melanoma, stage IV cTx N3 M1d(0)	61 d	75 d	Lichenoid inflammation	none	mIHC
**24**	male	64	Mono Anti-PD-1	Pembrolizumab	Metastatic melanoma, stage IIIC (pT4b, N1a (1/1sn), M0)	317 d	317 d	Lichenoid dermatitis, compatible with lichenoid drug exanthema in the corresponding clinic	none	mIHC

Supplementary Table S2. Dilution of antibodies and assigned Opal fluorophores.

**Opal**	**T celi panel**		**Macrophage panel**	
	antibody	dilution	antibody	dilution
480	CD8	1:200	pSTAT1	1:50
520	FoxP3	1 :100		
570	IL17A	1:200	MPO	1:150
620	Granzyme B	1 :100	c-Maf	1:50
690	PanCK	1 :100	PanCK	1:100
780	CD4	1 :100	CD68	1:100
